# Efficacy of intramuscular hydroxocobalamin supplementation in cats with cobalamin deficiency and gastrointestinal disease

**DOI:** 10.1111/jvim.15865

**Published:** 2020-08-20

**Authors:** Peter H. Kook, Roger H. Melliger, Martin Hersberger

**Affiliations:** ^1^ Clinic for Small Animal Internal Medicine, Vetsuisse Faculty University of Zurich Zurich Switzerland; ^2^ Melliger Analytics Lenzerheide Switzerland; ^3^ Department of Clinical Chemistry University Children's Hospital Zurich Zurich Switzerland

**Keywords:** biochemical, enteropathy, feline, lymphoma, vitamin B12

## Abstract

**Background:**

In humans, absorption and tissue retention rates of intramuscularly administered hydroxocobalamin (OH‐Cbl) are superior compared to cyanocobalamin (CN‐Cbl). Supplementation with OH‐Cbl has not been described in cats.

**Objectives:**

To evaluate effects of parenteral OH‐Cbl supplementation on clinical signs, serum Cbl and methylmalonic acid (MMA) concentrations in hypocobalaminemic cats with gastrointestinal disease.

**Animals:**

Twenty‐three client‐owned cats.

**Methods:**

Prospective study. Serum Cbl and MMA concentrations were determined at enrollment (t0), immediately before the 4th OH‐Cbl IM injection (300 μg, given q2 weeks) (t1), and 4 weeks after the 4th injection (t2). Severity of clinical signs (activity, appetite, vomiting, diarrhea, body weight) was graded at each time point and expressed as clinical disease activity score.

**Results:**

Median clinical disease activity score decreased significantly from t0 (6; range, 2‐10) to t1 (1; range, 0‐6) and t2 (1; range, 0‐9). Median serum Cbl concentration increased significantly from 111 pmol/L (range, 111‐218; reference range, 225‐1451 pmol/L) at t0 to 1612 pmol/L (range, 526‐14 756) (*P* < .001) at t1, and decreased again significantly to 712 pmol/L (range, 205‐4265) (*P* < .01) at t2. Median baseline serum MMA concentration at t0 (802 nmol/L; range, 238‐151 000; reference range, 120‐420 nmol/L) decreased significantly (*P* < .001) to 199 nmol/L (range, 29‐478) at t1, and was 205 nmol/L (range, 88‐734) at t2. Serum MMA concentrations normalized in 22/23 cats at t1, and were not significantly higher at t2 compared to t1.

**Conclusions and Clinical Importance:**

The herein described OH‐Cbl injection scheme appears efficacious for normalization of cellular Cbl deficiency in cats with gastrointestinal disease.

**Abbreviations**
Cbl: cobalamin
CN‐Cbl: cyanocobalamin
CV: coefficient of variation
MMA: methylmalonic acid
OH‐Cbl: hydroxocobalamin
SCL: small cell lymphoma

## 1 INTRODUCTION

Cobalamin (Cbl), also known as vitamin B_12_, is a water‐soluble B‐group vitamin and an essential cofactor for nucleic acid synthesis and hematopoiesis. Naturally occurring forms of B_12_ are methyl‐Cbl, adenosyl‐Cbl, and hydroxo‐Cbl (OH‐Cbl), whereas cyano‐Cbl (CN‐Cbl) is a synthetic B_12_ compound commonly used in supplements.^1^ Cbl absorption requires binding proteins and specific receptors along various parts of the gastrointestinal tract,^1^ and gastrointestinal disease can therefore lead to Cbl deficiency. In cell metabolism, adenosyl‐Cbl is needed as a cofactor for the conversion of methylmalonyl‐CoA to succinyl‐CoA via methylmalonyl‐CoA mutase and for remethylation of homocysteine via methionine synthase. In cats, Cbl deficiency causes a reduction in the activity of methylmalonyl‐CoA mutase, resulting in increases in serum methylmalonic acid (MMA) concentrations, but cats do not have increased homocysteine concentrations.^2^ Measurement of MMA allows assessment of availability of Cbl for cells and is considered the test of choice to detect cobalamin deficiency in people.^3^ Cats with undetectable or subnormal Cbl concentrations also have significantly increased MMA concentrations.^4^, ^5^, ^6^, ^7^Although it is not yet known when depletion of cellular Cbl stores and increases in MMA concentrations begin in the course of subnormal serum Cbl concentrations, MMA is currently considered the best indicator of Cbl status in cats.^2^, ^5^, ^6^, ^7^, ^8^, ^9^ Administration of parenteral Cbl to cats with subnormal Cbl values is currently considered a routine therapeutic procedure,^5^, ^6^, ^7^, ^8^ especially when daily oral Cbl supplementation is difficult to achieve.^10^ A widely followed recommendation suggests to administer 250 μg Cbl once weekly, for 6 weeks, followed by a dose 30 days later and determination of Cbl concentration 30 days after the last injection.^11^ At present, CN‐Cbl is generally recommended for routine usage in veterinary medicine, most probably because it is widely available and inexpensive.^11^ In cats, only the use of CN‐Cbl has been investigated so far,^4^, ^7^, ^8^ and it was recently shown that a Cbl supplementation scheme consisting of 6 weekly IM (250 μg) injections of CN‐Cbl failed to fully normalize serum and urine MMA concentrations in hypocobalaminemic cats with enteropathy.^7^ In people with various types of Cbl deficiencies, the natural‐occurring OH‐Cbl is considered the mainstay treatment and numerous studies consistently showed superior tissue retention rates of Cbl after supplementation with OH‐Cbl rather than CN‐Cbl together with increased urinary excretion of CN‐Cbl.^12^, ^13^, ^14^, ^15^, ^16^, ^17^, ^18^, ^19^, ^20^ In addition, IM injections of OH‐Cbl are thought to cause less pain than CN‐Cbl in people.^15^

For these reasons, we replaced CN‐Cbl with OH‐Cbl in our hospital in 2016 and this approach seemed to work well in our patients. Based on clinical experience as well as sporadic assessments of Cbl and MMA values, we decided to simplify our supplementation scheme from 6 weekly IM (250 μg) injections to 4 IM (300 μg) injections given every 2 weeks. The aim of the present study was therefore to evaluate the efficacy of intramuscularly administered OH‐Cbl on serum Cbl and MMA concentrations in cats with hypocobalaminemia and gastrointestinal disease before and after IM injections of OH‐Cbl given every 2 weeks for a total of 4 doses. A secondary aim was to assess the clinical benefits during OH‐Cbl supplementation.

## 2 MATERIALS AND METHODS

### 2.1 Animals and study design

This study was conducted at the Clinic for Small Animal Internal Medicine, Vetsuisse faculty, University of Zurich between September 2017 and November 2019. All cats were prospectively enrolled in the study based on the following criteria: a history of clinical signs compatible with gastrointestinal disease, which included diarrhea, vomiting, weight loss, anorexia, or weight loss with polyphagia; a serum Cbl concentration below the reference interval (225‐1451 pmol/L),^22^ and informed owner consent. Concurrent hyperthyroidism at inclusion was not considered an exclusion criterion when the cats' serum thyroxine concentration was within the reference range under treatment at enrollment and cats also exhibited ultrasonographic evidence of intestinal disease (generalized thickening of the muscularis layer of the small intestines)^23^ in addition to the abovementioned clinical signs.

All cats were supplemented with 4 IM injections of 300 μg OH‐Cbl (Vitarubin Depot, Streuli Pharma AG, 8730 Uznach, Switzerland) given every 2 weeks (q2 weeks). Baseline serum samples were collected before the first OH‐Cbl injection (t0) and Cbl and MMA were either measured directly at baseline, or serum aliquots were frozen at −80°C for later measurement of MMA concentration. Serum samples were again collected immediately before the 4th OH‐Cbl injection (t1), and 4 weeks after the 4th OH‐Cbl injection (t2) for measurement of serum Cbl concentration. Aliquots for later analysis of serum MMA concentration were directly frozen at −80°C. All stored samples for MMA measurements were shipped on dry ice to the laboratory, and shipping time did not exceed 20 minutes.

To assess the clinical status of cats undergoing OH‐Cbl supplementation, anamnestic and clinical findings from time points t0‐t1‐t2 were summarized and expressed as a clinical disease activity score, which ranged from 0 to 15. This score was based on the clinical signs used in a feline chronic enteropathy activity index^24^ and included level of activity, appetite, weight loss, vomiting, and diarrhea.^7^ The degree of severity for each clinical sign was scored as normal (0 points), mild (1 point), moderate (2 points), or severe (3 points). The study was approved by the Cantonal Veterinary Office of Zurich and conducted in accordance with guidelines established by the Animal Welfare Act of Switzerland.

### 2.2 Analyses

Serum Cbl concentration was measured with an automated competitive binding chemiluminescence assay (Immulite 2000, vitamin B_12_, Siemens Healthcare Diagnostics Inc, Newark, DE). The intra‐ and interassay coefficients of variation (CV) of the Cbl assay were 2.1% and 3.4%, respectively. The reference interval for serum Cbl was 225‐1452 pmol/L.^22^ Cbl concentrations at inclusion <111 pmol/L (below the detection limit) were truncated to 111 pmol/L for statistical analysis. All serum MMA concentrations were analyzed in the Division of Clinical Chemistry of the University Children's Hospital Zurich according to accredited methods. In brief, the samples were supplemented with an internal standard, precipitated, and analysis was done by ultraperformance liquid chromatography‐tandem mass spectrometry (UPLC‐TMS) on an Ultimate 3000 XRS UHPLC system (Dionex; Thermo Scientific) with a SCIEX5500 mass spectrometer (SCIEX, Framingham, Massachusetts) using multiple reaction monitoring. The lower limit of quantification for this method was 25 nmol/L. The interassay CV of the analyses were 5.8%. The reference intervals for serum MMA concentration was 120‐420 nmol/L.^7^

### 2.3 Statistical analysis

A Friedman‐test followed by a Nemenyi post hoc test were conducted to compare the effect of time on the repeated measures of serum Cbl, and serum MMA values measured at the 3 time points t0, t1, and t2. A Friedman‐test followed by a Nemenyi post hoc test was also used to assess changes over time in the clinical disease activity scores (t0‐t2). A Mann‐Whitney test was used to compare MMA values at baseline between polyphagic and nonpolyphagic cats. Statistical analyses and data visualization were done using statistical software (R statistical program for analyses with PMCMR and lme4 library) and graphing software (GraphPad Prism).

## 3 RESULTS

### 3.1 Animals

Breeds included European Shorthair (16/23), Balinese cat (1), British Longhair (1), Devon Rex (1), Egyptian Mau (1), Maine Coon (1), Norwegian forest cat (1), and Russian Blue (1). The median age was 12 years (range, 5 months to 18 years), median body weight was 3.7 kg (range, 1.62‐7.66), and median body condition score was 4 (2–5). Clinical signs present at the time of enrollment into the study were lethargy (19/23), weight loss (18/23), vomiting (15/23), small bowel diarrhea (15/23), inappetence or anorexia (12/23), and polyphagia (10/23). All 10 cats with subjectively increased appetite (polyphagia) had concurrent weight loss.

### 3.2 Clinicopathologic evaluation

After obtaining a clinical history, all cats underwent a physical examination and laboratory testing including a CBC, serum biochemistry panel, and urinalysis. Two cats were hyperthyroid and received medical treatment. Serum thyroxine concentration was measured in all 23 cats at baseline evaluation, and was well within the reference range in all cats. All cats were evaluated for pancreatitis using the DGGR‐lipase assay^25^; 9/23 cats had increased values. Two cats had minimally increased lipase activities (28, and 29 U/L; reference interval, 8‐26 U/L), the other 7 cats had a median value of 69 U/L (range, 43‐292 U/L) which was considered suggestive of pancreatitis.^26^ Serum trypsinogen‐like immunoreactivity (fTLI) was measured in 18 cats, and 1 cat was diagnosed with exocrine pancreatic insufficiency based on a fTLI value of 0.1 μg/L. All cats had been routinely dewormed (17/23) or had negative fecal analysis results (6/23).

Abdominal ultrasonography carried out in all 23 cats at the time of presentation showed abnormal intestinal findings in 22. Diffuse thickening of the muscularis layer of the small intestines was seen in 20, mesenteric lymphadenopathy in 14, and generalized hyperechoicity of the mucosal layer of the small intestines in 2 of the 23 cats. Histologic examination of the intestines was available for 12 cats after collection of endoscopic (gastroduodenoscopy and ileocoloscopy) biopsy specimens (7), full‐thickness biopsy specimens during surgery (4), or both specimens during postmortem examination (2).

### 3.3 Definitive and tentative diagnoses

A definitive (ie, histologic) diagnosis of gastrointestinal tract disease was available in 16 of 23 cats and comprised intestinal small cell lymphoma (8), inflammatory enteropathy (4; all lymphoplasmacytic enteritis), cholangitis/cholangiohepatitis (2; both cats also had suspected chronic enteropathy based on a thickened lamina muscularis propria), pancreatitis (1; well‐documented intra vitam, and confirmed on later postmortem examination together with lymphoplasmacytic enteritis and lymphoplasmacytic cholangiohepatitis), and exocrine pancreatic insufficiency (1). Chronic enteropathy was suspected in the 7 remaining cats based on clinical signs, low serum Cbl concentration, and compatible ultrasonographic features of the intestines, but was not confirmed histologically. Concurrent hyperthyroidism was known for 2 cats (1 with a clinical diagnosis of chronic enteropathy, 1 with small cell lymphoma). One cat had concurrent stage 2 chronic kidney disease (serum creatinine, 180 μmol/L; reference range, 98‐163 μmol/L).

### 3.4 Treatment

Treatments in addition to OH‐Cbl supplementation varied among cats and included dietary changes (17/23), prednisolone (15/23), probiotics (9/23), chlorambucil (7/23), antibiotics (2/23), and transdermal methimazole^27^ (2).

### 3.5 Concentrations of serum Cbl, and serum MMA

**FIGURE 1:**
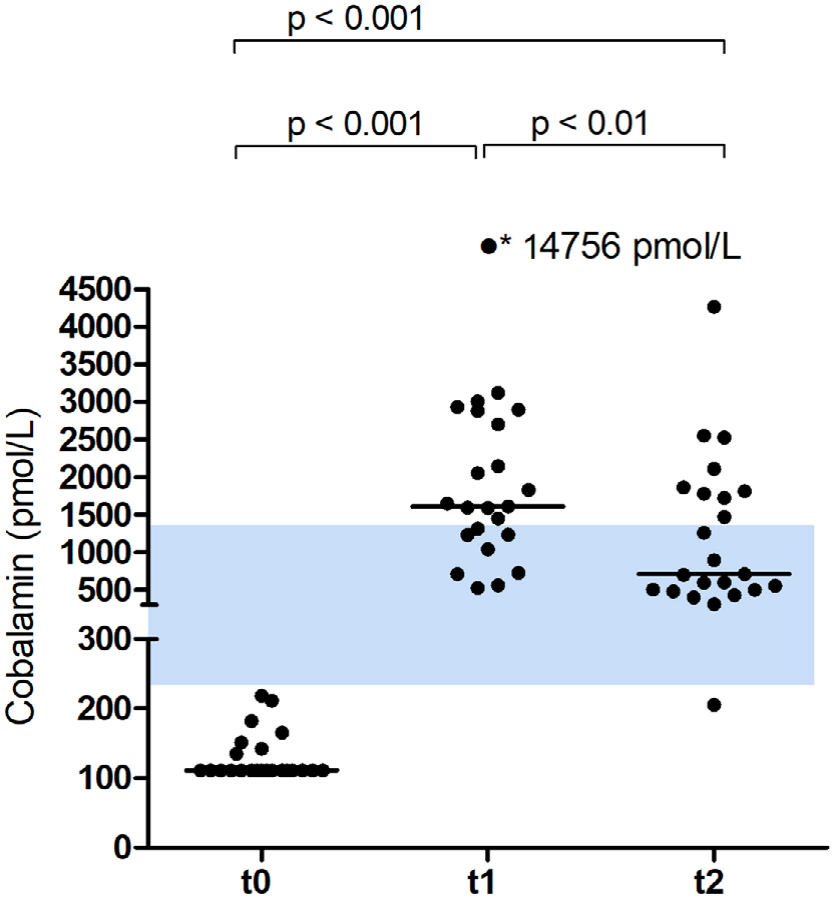
Scatter plot for serum Cbl concentration at baseline (t0), directly before the 4th IM injection of 300 μg OH‐Cbl given q2 weeks (t1), and 4 weeks after the 4th injection (t2); bars represent median values. At t2, the asterisk denotes an outlier with 14 756 pmol/L

At baseline, 16/23 cats had undetectable serum Cbl concentrations <111 pmol/L (reference range, 225‐1452 pmol/L), and 7 cats had subnormal values (median 165 pmol/L; range, 135‐218). Serum Cbl concentration (Figure 1) differed significantly over time (χ^2^ 44.087, *P* < .001). There was a significant increase (*P* < .001) in the median serum Cbl concentration from 111 pmol/L (range, 111‐218) at t0 to 1612 pmol/L (range, 526‐14 756) at t1, followed by a significant (*P* < .01) decrease to 712 pmol/L, range, 205‐4265) 4 weeks after the end of supplementation (t2).

At baseline, 20/23 cats had serum MMA concentrations above the reference range (120‐420 nmol/L). The corresponding MMA and Cbl values of the 3 cats with normal serum MMA concentrations were the following: 238 nmol/L and <111 pmol/L, 336 nmol/L and 165 pmol/L, and 403 nmol/L and 151 pmol/L).

**FIGURE 2:**
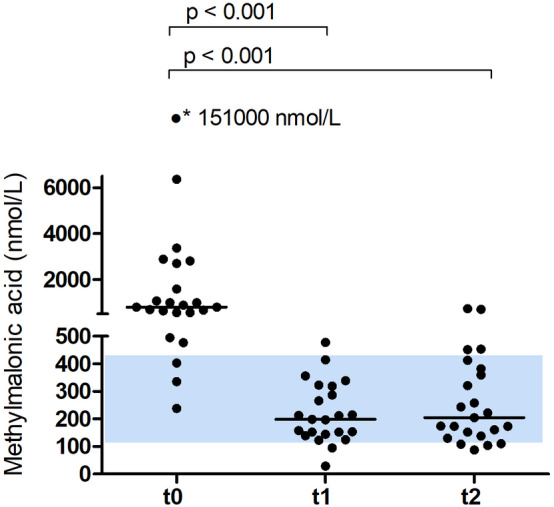
Scatter plot for serum MMA concentration at baseline (t0), directly before the 4th IM injection of 300 μg OH‐Cbl given q2 weeks (t1), and 4 weeks after the 4th injection (t2); bars represent median values. At t0, the asterisk denotes an outlier with 151 000 nmol/L

Serum MMA concentration (Figure 2) also differed significantly over time (χ^2^ 34.615, *P* < .001). The post hoc analysis showed that the median serum MMA concentration decreased significantly (*P* < .001) from 802 nmol/L (range, 238‐151 000) at t0 to 199 nmol/L (range, 29‐478) at t1. All but 1 cat had serum MMA concentrations in the reference range at t1. Median MMA concentrations (205 nmol/L; range, 88‐734) 4 weeks after the last Cbl injection (t2) did not differ significantly from t1. Cats presenting with polyphagia and weight loss (n = 10) had significantly (*P* = .04) higher median serum MMA concentrations (1291 nmol/L) compared to 13 cats without polyphagia (670 nmol/L).

### 3.6 Clinical signs

**FIGURE 3:**
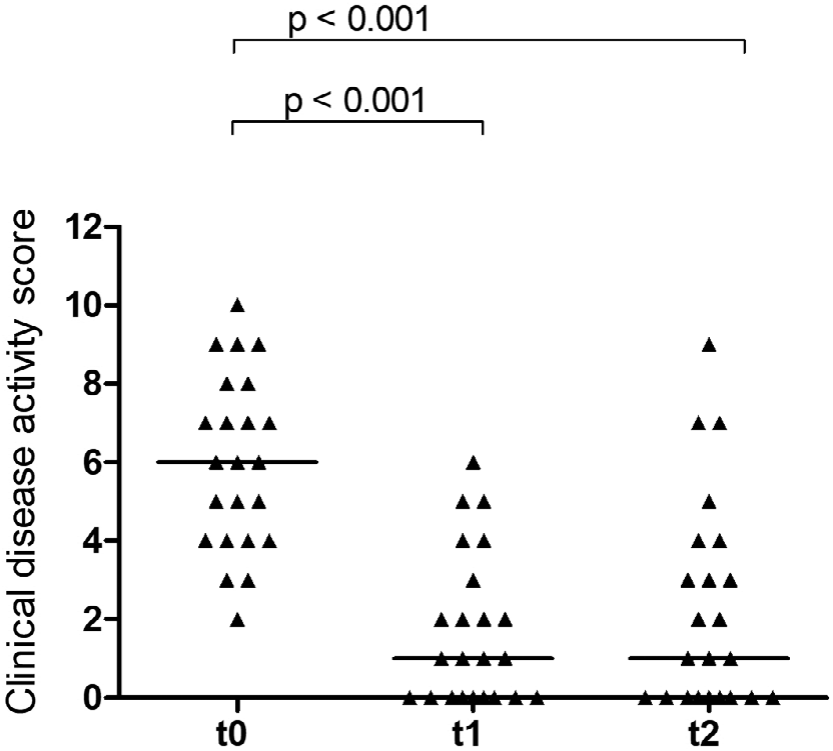
Scatter plot for the clinical disease activity score at baseline (t0), directly before the 4th IM injection of 300 μg OH‐Cbl given q2 weeks (t1), and 4 weeks after the 4th injection (t2); bars represent median values

The clinical disease activity scores (Figure 3) differed significantly over time (χ^2^ 24.617, *P* < .001). The median clinical disease activity score was 6 (range, 2‐10) at t0 and decreased significantly (*P* < .001) to 1 (range, 0‐6) at t1, and remained at 1 (range, 0‐9) 4 weeks later (t2).

## 4 DISCUSSION

In cats with Cbl deficiency and signs of gastrointestinal disease, 3 IM injections of 300 μg OH‐Cbl given every 2 weeks, significantly increased serum Cbl concentrations to supranormal values at t1, followed by a significant decrease to a median Cbl concentration within the reference range 4 weeks after the 4th injection. The median concentration of serum MMA decreased significantly into the reference range at t1, and results 4 weeks later at t2 did not differ significantly from t1. At t1, only 1/23 cats had a mildly increased serum MMA concentration (478 nmol/L), and this cat (diagnosis SCL) also had the highest serum MMA concentration (734 nmL/L) measured at t2. The combination of reduced frequency of required injections and superior posttreatment MMA concentrations highlight the advantages of OH‐Cbl over CN‐Cbl.

It has traditionally been recommended that serum Cbl concentration should be above the reference range at the time of retesting, and if supranormal serum cobalamin concentrations have not been achieved to continue supplementation every 2‐4 weeks.^11^ Our data at t1 and t2 do not support a strict need for supranormal serum Cbl results at retesting as MMA concentrations did not differ between both time points, even though median serum Cbl concentration had decreased again into the reference range. The results of the clinical disease activity score (significant decrease from t0 to t1 and no further change at t2) support this assumption, even if clinical signs must be interpreted with caution as all cats had also received additional treatment.

To our knowledge, supplementation with OH‐Cbl has not been reported before in cats. In a previous study assessing a supplementation scheme with 6 weekly IM CN‐Cbl injections in 20 cats with chronic enteropathy, serum and urine MMA concentrations had not fully normalized in 12/20 and 6/20 cats after 5 injections.^7^ Besides, the median serum Cbl concentration after 6 weekly CN‐Cbl injections was lower (612 pmol/L) compared to 4 weeks after 4 OH‐Cbl injections given q2 weeks in this study (712 pmol/L).^7^

Although varied concurrent treatments could have also influenced our results, we presume these differences in outcome might relate to the Cbl formulation. It has been shown experimentally in dogs that only a minor part of the injected dose of CN‐Cbl is eventually utilized, whereas a large percentage is being lost in the urine during the first hours after injection.^18^ When the same dogs received OH‐Cbl, higher serum binding and more prolonged increases in serum Cbl concentrations and lower urinary excretion was noted compared with CN‐Cbl.^18^ It was speculated that the difference in serum binding may lie in the chemical stability of CN‐Cbl, which limits the sites at which it may be bound as compared to OH‐Cbl which readily dissociated and may equilibrate with a variety of serum constituents.^17^ Possible differences in protein binding likely have also implications for cellular markers of Cbl availability as only Cbl bound to transcobalamin is available to cells.^30^

Superior bioavailability of OH‐Cbl over CN‐Cbl has also been proven in humans. Slower disappearance of OH‐Cbl from site of injection, increased liver uptake, and less rapid urinary excretion when compared with CN‐Cbl were demonstrated by various authors.^14^, ^15^, ^16^, ^17^, ^18^, ^19^, ^20^, ^28^ Hertz et al reported that after IM injection of 1 mg CN‐Cbl and OH‐Cbl, healthy individuals excreted within 24 hours about 80% and about 25%, respectively, in the urine.^15^ Further dialysis experiments showed that OH‐Cbl passed more slowly through membranes than does CN‐Cbl, and that OH‐Cbl is bound to serum proteins in far greater quantities than is CN‐Cbl.^15^ These observations were confirmed in long‐term studies when humans with pernicious anemia were given equal amounts of either OH‐Cbl or CN‐Cbl IM injections,^14^, ^17^ and similar results have been published when comparing oral OH‐Cbl to CN‐Cbl.^29^ Against this background, it is interesting to note that it has also been mentioned in small animal medicine that CN‐Cbl might fail to increase serum Cbl concentrations for reasons currently not understood, and it has been speculated that OH‐Cbl might instead be more effective in these patients.^11^

Superior bioavailability of OH‐Cbl in humans may be because of different affinities for the blood‐transport binding proteins, cell receptors for Cbl uptake, and intracellular enzymes involved in their conversion to intracellular cobalamin.^13^ The latter could have been the reason that CN‐Cbl injections in humans with an inherited disorder of intracellular Cbl metabolism were inadequate, and biochemical (ie, MMA, homocysteine) as well as clinical parameters normalized only after patients were switched to OH‐Cbl injections.^21^ Unfortunately, very little is known about Cbl‐binding proteins in cats,^31^ and studies on cellular uptake and activation of intracellular methylmalonyl‐CoA mutase have not been carried out. Our serum MMA results at t1 and t2 basically agree with the concept of improved cellular uptake of OH‐Cbl when comparing it to MMA results after supplementation with CN‐Cbl.^7^ However, differences in the study designs hinder direct comparisons of results. Instead of 6 weekly injections, 4 injections given q2 weeks were administered in the present study and also the dosage per IM injection was increased from 250 to 300 μg. The normalized postsupplementation Cbl and MMA values in our study (t1) therefore reflect the effects of 3 OH‐Cbl 300 μg injections, whereas the results of the follow‐up measurement reflect the effect of 4 OH‐Cbl 300 μg injections. The rationale behind this simplified approach was to facilitate the whole Cbl supplementation process for our patients. Our previous clinical experience with OH‐Cbl in cats let us to believe that cats would similar to humans also have better bioavailability of OH‐Cbl. A positive side‐effect of the shortened supplementation was that we could spare our patients hospital visits which improved overall compliance. It had taken 4 years to include 20 cats for our previous study,^7^ and almost all of the participating cat owners described the supplementation protocol of 6 weekly injections and additional rechecks as cumbersome.

Cats displaying a combination of polyphagia and weight loss had significantly higher serum MMA concentrations compared to cats without polyphagia. This comparison could not be made for serum Cbl as almost all results were <111 pmol/L. Polyphagia with concurrent weight loss is a hallmark clinical sign of malabsorption and usually regarded as an indicator of clinical disease severity. In the future, MMA might be a biomarker for disease severity in cats with gastrointestinal disease, however at least at this point the cost of analysis complicates routine use.

Our study had several limitations. One limitation was the inclusion of cats with more than 1 clearly defined disease process as well as with different stages and severity of disease. However, we believe this was unavoidable. Even if only cats with 1 diagnosis (eg, intestinal SCL) had been included, it would have been impossible to guarantee uniform spatial distribution of disease among patients. That means we could not control for individual interference with renewed absorption of biliary Cbl during enterohepatic circulation. Another limitation was that our modified injection scheme made direct comparisons of OH‐Cbl versus CN‐Cbl supplementation difficult. Furthermore, concurrent individual treatments made it difficult to accurately assess the magnitude of the beneficial clinical effect of OH‐Cbl supplementation, but we could not justify withholding necessary medical treatments. The magnitude of remission of clinical signs at t1 (1; range, 0‐6) was the same compared to the identical score used in the previous study with CN‐Cbl,^7^ but also in the previous study most cats received additional treatments.

In conclusion, our findings show that 4 injections of OH‐Cbl are efficacious for normalization of serum Cbl and cellular Cbl deficiency in cats with hypocobalaminemia and gastrointestinal disease. The beneficial metabolic effects appear to be greater when compared to previously published results with CN‐Cbl.^7^ Comparative studies on the optimal Cbl formulation (OH‐Cbl versus CN‐Cbl) are needed in cats with Cbl deficiency.

## CONFLICT OF INTEREST DECLARATION

Authors declare no conflict of interest.

## OFF‐LABEL ANTIMICROBIAL DECLARATION

Authors declare no off‐label use of antimicrobials.

## INSTITUTIONAL ANIMAL CARE AND USE COMMITTEE (IACUC) OR OTHER APPROVAL DECLARATION

Approved by the Cantonal Veterinary Office of Zurich and conducted in accordance with guidelines established by the Animal Welfare Act of Switzerland.

## HUMAN ETHICS APPROVAL DECLARATION

Authors declare human ethics approval was not needed for this study.
